# Supplementary material for: Survival of vascularized osseous flaps in mandibular reconstruction: A network meta-analysis
Source: PLoS One. 2021 Oct 22;16(10):e0257457. doi: 10.1371/journal.pone.0257457 (PMC8535428; doi:10.1371/journal.pone.0257457)
Supplement: S1 Fig — (PDF) [file pone.0257457.s001.pdf]

Which vascularized osseous flap is associated with the highest survival for mandibular reconstruction? A network meta-analysis

Figure S1

Forest plot result of pairwise meta-analysis of direct comparison of different osseous free flap for mandibular reconstruction.

FFF versus DCIA

Meta Analysis

Statistics for each study

Study name	Odds ratio	Lower limit	Upper limit	Z-Value	p-Value
Haughey et al. 1994	8.333	0.351	198.086	1.312	0.190
Heller et al. 1995	11.000	0.380	318.606	1.396	0.163
Shpitzer et al. 1985	1.761	0.401	7.735	0.749	0.454
Takushima et al. 2001	0.862	0.237	3.138	-0.225	0.822
Chang et al. 2001	0.094	0.006	1.393	-1.719	0.086
Yilmaz et al. 2008	0.580	0.022	15.265	-0.326	0.744
Van Germert et al. 2011	0.088	0.004	1.919	-1.545	0.122
Hanken et al. 2014	0.673	0.024	18.845	-0.233	0.816
T. Wilkman, et al. 2018	0.546	0.063	4.747	-0.548	0.584
Ritschl et al. 2020	2.579	0.134	49.573	0.628	0.530
Schultz et al. 2015	0.892	0.032	25.147	-0.067	0.946
Chen et al. 1994	0.512	0.020	13.191	-0.404	0.686
Politi,and Toro. 2012	3.857	0.142	104.645	0.802	0.423
	0.980	0.505	1.902	-0.060	0.953

Odds ratio and 95% CI

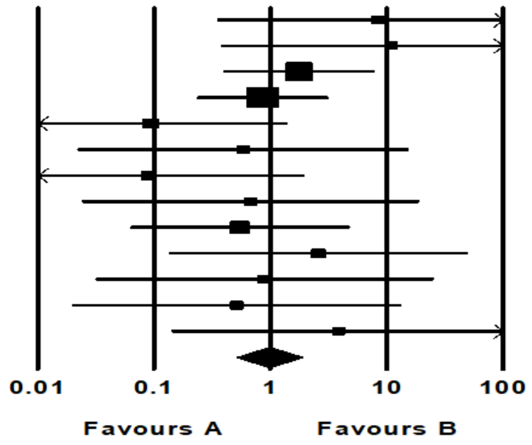

Meta Analysis

DCIA versus scapula

Meta Analysis

Study name	Statistics for each study				
	Odds ratio	Lower limit	Upper limit	Z-Value	p-Value
Takushima et al. 2001	4.900	0.928	25.862	1.872	0.061
T. Wilkman, et al. 2018	7.443	0.412	134.380	1.360	0.174
	5.436	1.285	22.995	2.301	0.021

Odds ratio and 95% CI

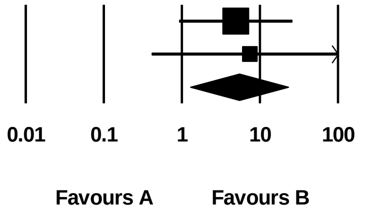

Meta Analysis

FFF versus scapula

Meta Analysis

Study name	Statistics for each study					Odds ratio and 95% CI
	Odds ratio	Lower limit	Upper limit	Z-Value	p-Value	
Takushima et al. 2001	4.224	0.769	23.190	1.658	0.097	
Dowthwaite et al. 2013	0.311	0.012	7.786	-0.711	0.477	
T. Wilkman, et al. 2018	5.571	0.215	144.112	1.035	0.301	
	2.774	0.707	10.876	1.464	0.143	

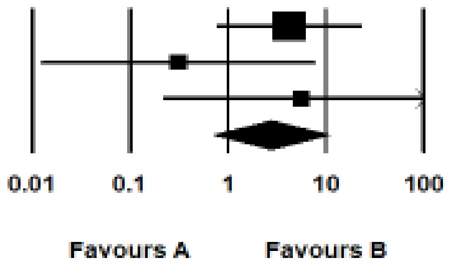

Meta Analysis

DCIA versus ORFF

Meta Analysis

Study name	Statistics for each study				
	Odds ratio	Lower limit	Upper limit	Z-Value	p-Value
Boyd et al.	1.643	0.080	33.741	0.322	0.747
	1.643	0.080	33.741	0.322	0.747

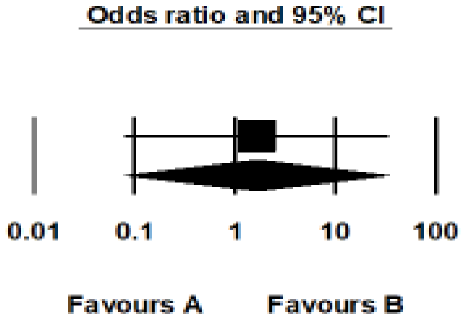

Meta Analysis
