# Supplementary figures and images for: Survival of vascularized osseous flaps in mandibular reconstruction: A network meta-analysis
Source: PLoS One. 2021 Oct 22;16(10):e0257457. doi: 10.1371/journal.pone.0257457 (PMC8535428; doi:10.1371/journal.pone.0257457)

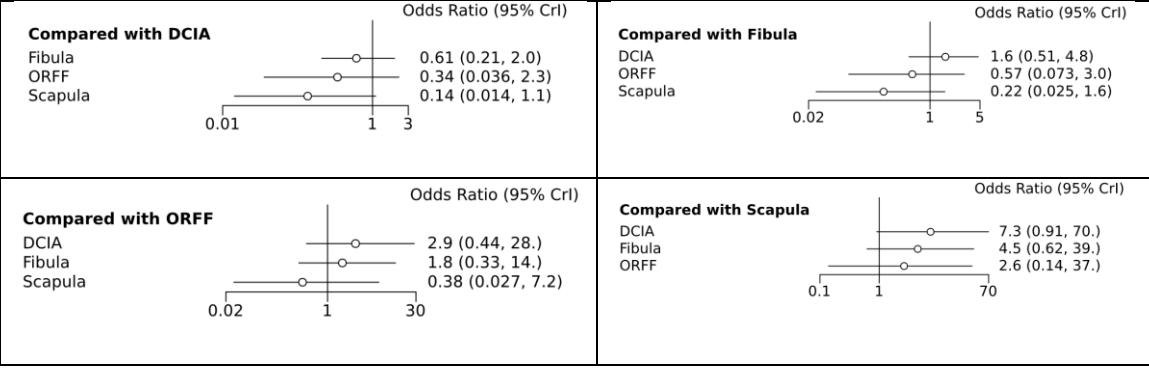

Supplement: S2 Fig — (PDF) [file pone.0257457.s002.pdf]
