# Supplementary material for: Survival of vascularized osseous flaps in mandibular reconstruction: A network meta-analysis
Source: PLoS One. 2021 Oct 22;16(10):e0257457. doi: 10.1371/journal.pone.0257457 (PMC8535428; doi:10.1371/journal.pone.0257457)
Supplement: S2 Table — (DOCX) [file pone.0257457.s004.docx]

|  | Search terms | No. |
| --- | --- | --- |
| Pubmed  28-03-2021 | (((((((mandibl*) ) OR (oral cancer[MeSH Terms])) OR (bone tissue neoplasms[MeSH Terms])) OR (mandibulectomy)) AND ((((free flap[MeSH Terms]) OR (free flaps, microsurgical[MeSH Terms])) OR (osseous flap)) OR (bony flap))) AND (((((((fibula[MeSH Terms]) OR (scapula[MeSH Terms])) OR (radius[MeSH Terms])) OR (DCIA FLAP)) OR (osteocutaneous fibula flap)) OR (deep circumflex iliac artery)) OR (radialforearm flap))) AND (((survival rate[MeSH Terms]) OR (survival rates[MeSH Terms])) OR (flap failure)) | 87 |
| SCOPUS  28-03-2021 | ( bone AND tissue AND neoplasms OR mandibulectomy ) AND ALL ( free AND flap OR osseous AND flap ) AND ALL ( fibula OR scapula OR radius OR fibula AND flap OR deep AND circumflex AND iliac AND artery AND flap OR forearm AND flap ) AND ALL ( flap AND survival ) OR TITLE-ABS-KEY ( flap AND failure ) ) | 83 |
| EMBASE  28-03-2021 | (((mandible OR 'mandible resection' OR 'bone defect') AND 'free flap reconstruction' OR 'bone graft'/exp OR 'bone graft') AND fibula OR scapula OR 'deep circumflex iliac artery flap' OR radius OR 'radial forearm flap') AND 'flap survival' | 157 |
| Cochran  28-03-2021 | #1 mandible #2 mouth tumor #3 mandible resection #4 bone defect  #5 # 1 OR #2 OR #3 OR #4  #6 free tissue flap #7 flap reconstruction #8 bone graft  #9 #6 OR #7 OR #8  #10 fibula #11 Scapula #12 deep circumflex iliac artery #13 radius  #14 radial forearm flap  #15 #10 OR #11 OR #12 OR #13 OR #14  #16 flap survival  #17 #5 AND #9 AND #15 AND #16 | 9 |
