# Supplementary material for: Survival of vascularized osseous flaps in mandibular reconstruction: A network meta-analysis
Source: PLoS One. 2021 Oct 22;16(10):e0257457. doi: 10.1371/journal.pone.0257457 (PMC8535428; doi:10.1371/journal.pone.0257457)
Supplement: S3 Table — (DOCX) [file pone.0257457.s005.DOCX]

Table S2

Risk of bias assessment

| Author | selection | comparability | outcomes | Total |
| --- | --- | --- | --- | --- |
| Ritschl et al 2020 | ** | * | *** | ****** |
| Haughey1994 | *** | * | *** | ******* |
| Heller et al 1995 | ** | * | ** | ***** |
| Schultz et al. 2015 | *** | * | *** | ******* |
| Shpitzer et al, 1999 | ** | ** | *** | ******* |
| Takushima et al 2001 | ** | ** | *** | ******* |
| Chen et al 2014 | ** | ** | ** | ****** |
| Wilkman et al 2018 | ** | ** | *** | ******* |
| Hanken et al 2014 | ** | * | ** | ***** |
| Deleyiannis et al 2006 | ** | ** | *** | ******* |
| Dowthwaite et al 2013 | ** | * | *** | ****** |
| Fujiki et al 2013 | ** | ** | *** | ******* |
| Boyd et al 1990 | *** | ** | ** | ******* |
| Dean et al 2011 | ** | ** | *** | ******* |
| van Gemert et al 2012 | ** | ** | *** | ******* |
| Chang et al 2001 | * | ** | ** | ***** |
| Virgin et al 2010 | ** | ** | *** | ******* |
| Yilmaz et al 2008 | ** | ** | ** | ****** |
| Hanken et al. 2014 | * | ** | ** | ***** |
| Chen et al 1994 | * | ** | ** | ***** |
| Yu et al. 2019 | ** | ** | ** | ****** |
| Winters et al.2006 | ** | ** | ** | ****** |

(0 to 3 stars to represent high risk of bias; 4 to 6 stars, moderate risk of bias; and 7 to 9 stars, low risk of bias)

Seven studies with moderate risk of bias, and 10 with low risk of bias.
