# Supplementary material for: Survival of vascularized osseous flaps in mandibular reconstruction: A network meta-analysis
Source: PLoS One. 2021 Oct 22;16(10):e0257457. doi: 10.1371/journal.pone.0257457 (PMC8535428; doi:10.1371/journal.pone.0257457)
Supplement: S4 Table — (DOCX) [file pone.0257457.s006.docx]

|  | Study | Reason(s) for exclusion |
| --- | --- | --- |
| 1 | Möhlhenrich et al. 2016 [1] | Flap survival rate was not reported. |
| 2 | Okcu et al. 2018 [2] | Flap survival rate was not reported. |
| 3 | González-García et al. 2018[3] | Some of the patients in the group of the radial forearm underwent soft tissue reconstruction without bone repair. |
| 4 | García-Díez et al. 2013[4] | Only one patient underwent fibula flap. |
| 5 | Syczewska et al. 2018[5] | Flap survival rate was not reported. |
| 6 | Chiapasco et al. 2000 [6] | Flap survival rate was not reported and the number of patients in fibula flap group was less than 5 patients. |
| 7 | Mertens et al. 2014[7] | Flap survival rate was not reported. |
| 8 | Gabr et al. 2004[8] | Two flaps Iliac along with Ulnar flap were used together. |
| 9 | Wilkman et al. 2017 [9] | Flap survival rate was not reported. |
| 10 | Kniha et al. 2017 [10] | Flap survival rate was not reported. |
| 11 | Disa et al. 1990 [11] | Flap survival rate was not reported. |
| 12 | Ricotta et al. 2019 [12] | The number of patients in each group was less than 5 patients. |
| 13 | Urken et al. 1998 [13] | Survival rate for each group was not reported. |
| 14 | Militsakh et al. 2005 [14] | The participants in the groups 2 underwent fibula or scapula, so the number of the lost flaps was unclear. |
| 15 | Daniel et al. 2000[15] | The number of the included participants in each group was less than 5 |
| 16 | Bianchi et al. 2013 [16] | Flap survival rate was not reported. |
| 17 | Ling et al. 2013 [17] | Flap survival rate was not reported. |
| 18 | Swendseid et al. 2019 [18] | The flap survival rate of each group was not reported. |
| 19 | Coleman et al. 2000 [19] | Flap survival rate was not reported. |
| 20 | Van Genechten et al. 2015 [20] | The authors did not clearly mention how many flaps lost in each group. |
| 21 | Valerio et al. 2015 [21] | The number of the included participants in each group was less than 5 |
| 22 | Hirsch et al. 2008[22] | Only on patient was in the DCIA group. |
| 23 | Lou et al. 2019 [23] | Flap failure was reported but it was unclear how many flaps lost in each groups |

**References**

[1] Möhlhenrich SC, Kniha K, Elvers D, Ayoub N, Goloborodko E, Hölzle F, et al. Intraosseous stability of dental implants in free revascularized fibula and iliac crest bone flaps. J Cranio-Maxillofacial Surg 2016;44:1935–9. https://doi.org/10.1016/j.jcms.2016.09.011.

[2] Okcu Y, Rustemeyer J. Continuity defects of the mandible: Comparison of three techniques for osseous reconstruction and their impact on implant loading. J Cranio-Maxillofacial Surg 2018;46:858–67. https://doi.org/10.1016/j.jcms.2018.03.001.

[3] González-García R, Naval-Gías L, Rodríguez-Campo FJ, Román-Romero L. Reconstruction of Oromandibular Defects by Vascularized Free Flaps: The Radial Forearm Free Flap and Fibular Free Flap as Major Donor Sites. J Oral Maxillofac Surg 2009;67:1473–7. https://doi.org/10.1016/j.joms.2006.06.286.

[4] García-Díez EM, Cho-Lee GY, Raigosa-García JM, Sieira-Gil R, Pagès CM. Rhytidectomy approach for mandibular reconstruction with microvascular free flaps after resection of mandibular benign tumors. J Oral Maxillofac Surg 2013;71:2156–68. https://doi.org/10.1016/j.joms.2013.05.009.

[5] Syczewska M, Krajewski R, Kirwil M, Szczerbik E, Kalinowska M. Gait changes in patients after reconstruction of facial bones with fibula and iliac crest free vascularized flaps. Acta Bioeng Biomech 2018;20:185–90. https://doi.org/10.5277/ABB-01062-2017-02.

[6] Chiapasco M, Abati S, Ramundo G, Rossi A, Romeo E, Vogel G. Behavior of implants in bone grafts or free flaps after tumor resection. Clin Oral Implants Res 2000;11:66–75. https://doi.org/10.1034/j.1600-0501.2000.011001066.x.

[7] Mertens C, Decker C, Engel M, Sander A, Hoffmann J, Freier K. Early bone resorption of free microvascular reanastomized bone grafts for mandibular reconstruction - A comparison of iliac crest and fibula grafts. J Cranio-Maxillofacial Surg 2014;42. https://doi.org/10.1016/j.jcms.2013.08.010.

[8] Gabr E, Kobayashi MR, Salibian AH, Armstrong WB, Sundine M, Calvert JW, et al. Mandibular Reconstruction: Are Two Flaps Better Than One? Ann Plast Surg 2004;52:31–5. https://doi.org/10.1097/01.sap.0000099819.90674.c6.

[9] Wilkman T, Apajalahti S, Wilkman E, Törnwall J, Lassus P. A Comparison of Bone Resorption Over Time: An Analysis of the Free Scapular, Iliac Crest, and Fibular Microvascular Flaps in Mandibular Reconstruction. J Oral Maxillofac Surg 2017;75:616–21. https://doi.org/10.1016/j.joms.2016.09.009.

[10] Kniha K, Möhlhenrich SC, Foldenauer AC, Peters F, Ayoub N, Goloborodko E, et al. Evaluation of bone resorption in fibula and deep circumflex iliac artery flaps following dental implantation: A three-year follow-up study. J Cranio-Maxillofacial Surg 2017;45:474–8. https://doi.org/10.1016/j.jcms.2017.01.014.

[11] Disa JJ, Hidalgo DA, Cordeiro PG, Winters RM TH. Evaluation of bone height in osseous free flap mandible reconstruction: an indirect measure of bone mass. 1990:103(5):1371-1377.

[12] Ricotta F, Battaglia S, Sandi A, Pizzigallo A, Marchetti C, Tarsitano A, et al. Use of a CAD-CAM inferior alveolar nerve salvage template during mandibular resection for benign lesions 2019:117–21. https://doi.org/10.14639/0392-100X-2408.

[13] M L Urken, D Buchbinder, P D Costantino, U Sinha, D Okay, W Lawson HFB. Oromandibular Reconstruction Using Microvascular Composite Flaps 1998;124:46–55.

[14] Oleg N. Militsakh, Andreas Werle, Nadia Mohyuddin, E. Bruce Toby, J. David Kriet, Derrick I. Wallace, Douglas A. Girod TTT. Comparison of Radial Forearm With Fibula and Scapula Osteocutaneous Free Flaps for Oromandibular Reconstruction 2005;131:571–5.

[15] Deschler DG, Hayden RE. The optimum method for reconstruction of complex lateral oromandibular-cutaneous defects. Head Neck 2000;22:674–9. https://doi.org/10.1002/1097-0347(200010)22:7<674::AID-HED6>3.0.CO;2-B.

[16] Bianchi B, Ferri A, Ferrari S, Leporati M, Copelli C, Ferri T, et al. Mandibular resection and reconstruction in the management of extensive ameloblastoma. J Oral Maxillofac Surg 2013;71:528–37. https://doi.org/10.1016/j.joms.2012.07.004.

[17] Ling XF, Peng X, Samman N. Donor-site morbidity of free fibula and DCIA flaps. J Oral Maxillofac Surg 2013;71:1604–12. https://doi.org/10.1016/j.joms.2013.03.006.

[18] Swendseid B, Kumar A, Sweeny L, Wax MK, Zhan T, Goldman RA, et al. Long-Term Complications of Osteocutaneous Free Flaps in Head and Neck Reconstruction 2019. https://doi.org/10.1177/0194599820912727.

[19] Coleman SC, Burkey BB, Day TA, Resser JR, Netterville JL, Dauer E, et al. Increasing use of the scapula osteocutaneous free flap. Laryngoscope 2000;110:1419–24. https://doi.org/10.1097/00005537-200009000-00001.

[20] Van Genechten MLV, Batstone MD. The relative survival of composite free flaps in head and neck reconstruction. Int J Oral Maxillofac Surg 2016;45:163–6. https://doi.org/10.1016/j.ijom.2015.09.022.

[21] Valerio I, Green JM 3rd, Sacks JM, Thomas S, Sabino J, Acarturk TO. Vascularized osseous flaps and assessing their bipartate perfusion pattern via intraoperative fluorescence angiography. J Reconstr Microsurg 2015;31:45–53. https://doi.org/10.1055/s-0034-1383821.

[22] Hirsch DL, Bell RB, Dierks EJ, Potter JK, Potter BE. Analysis of Microvascular Free Flaps for Reconstruction of Advanced Mandibular Osteoradionecrosis: A Retrospective Cohort Study. J Oral Maxillofac Surg 2008;66:2545–56. https://doi.org/https://doi.org/10.1016/j.joms.2007.08.041.

[23] Lou C, Yang X, Hu L, Hu Y, S.P. Loh J, Ji T, et al. Oromandibular reconstruction using microvascularized bone flap: report of 1038 cases from a single institution. Int J Oral Maxillofac Surg 2019;48:1001–8. https://doi.org/10.1016/j.ijom.2019.02.017.
